# Mitochondrial Lon is over-expressed in high-grade gliomas, and mediates hypoxic adaptation: potential role of Lon as a therapeutic target in glioma

**DOI:** 10.18632/oncotarget.12681

**Published:** 2016-10-15

**Authors:** Kaijun Di, Naomi Lomeli, Spencer D. Wood, Christopher D. Vanderwal, Daniela A. Bota

**Affiliations:** ^1^ Department of Neurological Surgery, University of California, Irvine, California, United States; ^2^ Department of Pathology and Laboratory Medicine, University of California, Irvine, California, United States; ^3^ Department of Chemistry, University of California, Irvine, California, United States; ^4^ Department of Neurology, University of California, Irvine, California, United States; ^5^ Chao Family Comprehensive Cancer Center, University of California, Irvine, California, United States

**Keywords:** Lon, glioma, hypoxia, Lon inhibitor

## Abstract

Mitochondrial dysfunction is a hallmark of cancer biology. Tumor mitochondrial metabolism is characterized by an abnormal ability to function in scarce oxygen conditions through glycolysis (the Warburg effect), and accumulation of mitochondrial DNA defects are present in both hereditary neoplasia and sporadic cancers. Mitochondrial Lon is a major regulator of mitochondrial metabolism and the mitochondrial response to free radical damage, and plays an essential role in the maintenance and repair of mitochondrial DNA. Despite these critical cellular functions of Lon, very little has been reported regarding its role in glioma. Lon expression in gliomas and its relevance with patient survival was examined using published databases and human tissue sections. The effect of Lon in glioma biology was investigated through siRNA targeting *Lon*. We also tested the *in vitro* antitumor activity of Lon inhibitor, CC4, in the glioma cell lines D-54 and U-251. High Lon expression was associated with high glioma tumor grade and poor patient survival. While Lon expression was elevated in response to a variety of stimuli, Lon knockdown in glioma cell lines decreased cell viability under normal conditions, and dramatically impaired glioma cell survival under hypoxic conditions. Furthermore, the Lon inhibitor, CC4, efficiently prohibited glioma cell proliferation and synergistically enhanced the therapeutic efficacy of the chemotherapeutic agents, temozolomide (TMZ) and cisplatin. We demonstrate that Lon plays a key role in glioma cell hypoxic survival and mitochondrial respiration, and propose Lon as a promising therapeutic target in the treatment of malignant gliomas.

## INTRODUCTION

Mitochondrial changes are postulated to represent a significant part of cancer cell biology. Cancer cells must survive and adapt to challenging microenvironments; notably in conditions in which tumor growth makes oxygen and glucose scarce [1]. These restrictive conditions require fundamental changes in cellular metabolism, including the respiratory transition from oxidative phosphorylation to glycolysis known as the Warburg effect [2]. Though it is well known that adaptation and survival in hypoxic conditions are hallmarks of aggressive tumors [3], little is known about the mitochondrial mechanisms involved in the hypoxic adaptation.

Glioblastoma (GBM), the most common primary neoplasm of the brain, is a very aggressive tumor with a historical survival of less than one year [4]. GBM can arise *de novo*, but can also result from the progression of low-grade astrocytomas. This malignant transformation is mediated by high expression of the transcriptional activator hypoxia-inducible factor 1α (HIF-1α), and hypoxia-induced phenotypic changes such as abnormal vascular proliferation and necrosis. In normal mammalian cells, HIF-1α functions as an oxygen sensor, regulating expression of multiple genes in response to oxygen availability [5]. Under hypoxic conditions, HIF-1α spearheads a switch from oxidative to glycolytic metabolism thus optimizing respiratory efficiency in cells [6]. Even though hundreds of genes are regulated by HIF-1α, mitochondrial Lon is one of the few genes known to be directly up-regulated by HIF-1α [6].

Lon is a highly evolutionarily conserved, ATP-stimulated protease [7], with homologues identified in bacteria [8, 9], yeast [10], and the mitochondria of mammals [11–13]. We previously reported that in normal mammalian cells *Lon* down-regulation leads to impaired mitochondrial proteolysis, accumulation of both native and oxidized aconitase [14], loss of mitochondrial DNA, and finally apoptotic cell death [15]. Mammalian Lon can also act as a chaperone, independent of its proteolytic activity [16], and it promotes the assembly [16] and degradation of*cytochrome c* oxidase (COX) subunits[6]. Its expression is required for the maintenance and repair of mitochondrial (*mt*) DNA [17]. Despite these critical cellular functions of Lon, little has been reported regarding its role in cancer biology. Similar to our previous publication on Lon inhibition ultimately leading to apoptotic cell death, other reports demonstrated that Lon knockdown in a lung cancer cell line [18] and lymphoma cells [19] also leads to cell death. Taken together, further exploration to elucidate the role(s) of Lon in tumor biology is clearly warranted.

In this study, we test the hypothesis that Lon plays an integral role in glioma tumor progression. The presented data demonstrates that human malignant glioma tissue and primary glioma cell cultures have elevated Lon levels, and those levels correlate with increased malignancy. We demonstrate that in malignant glioma cells, Lon is regulated by HIF-1α, it controls survival, and mediates adaption to hypoxia. Furthermore, we show that Lon inhibition significantly suppressed glioma cell proliferation and synergistically enhanced the therapeutic efficacy of both temozolomide (TMZ) and cisplatin. Understanding the relationship between tumor environmental changes, such as hypoxia, and mitochondrial biology can open the door for designing new treatments targeting the cancer cell mitochondria.

## RESULTS

### Lon over-expression is observed in glioblastoma tumor tissues and corresponds to decreased patient survival

**Figure 1:**
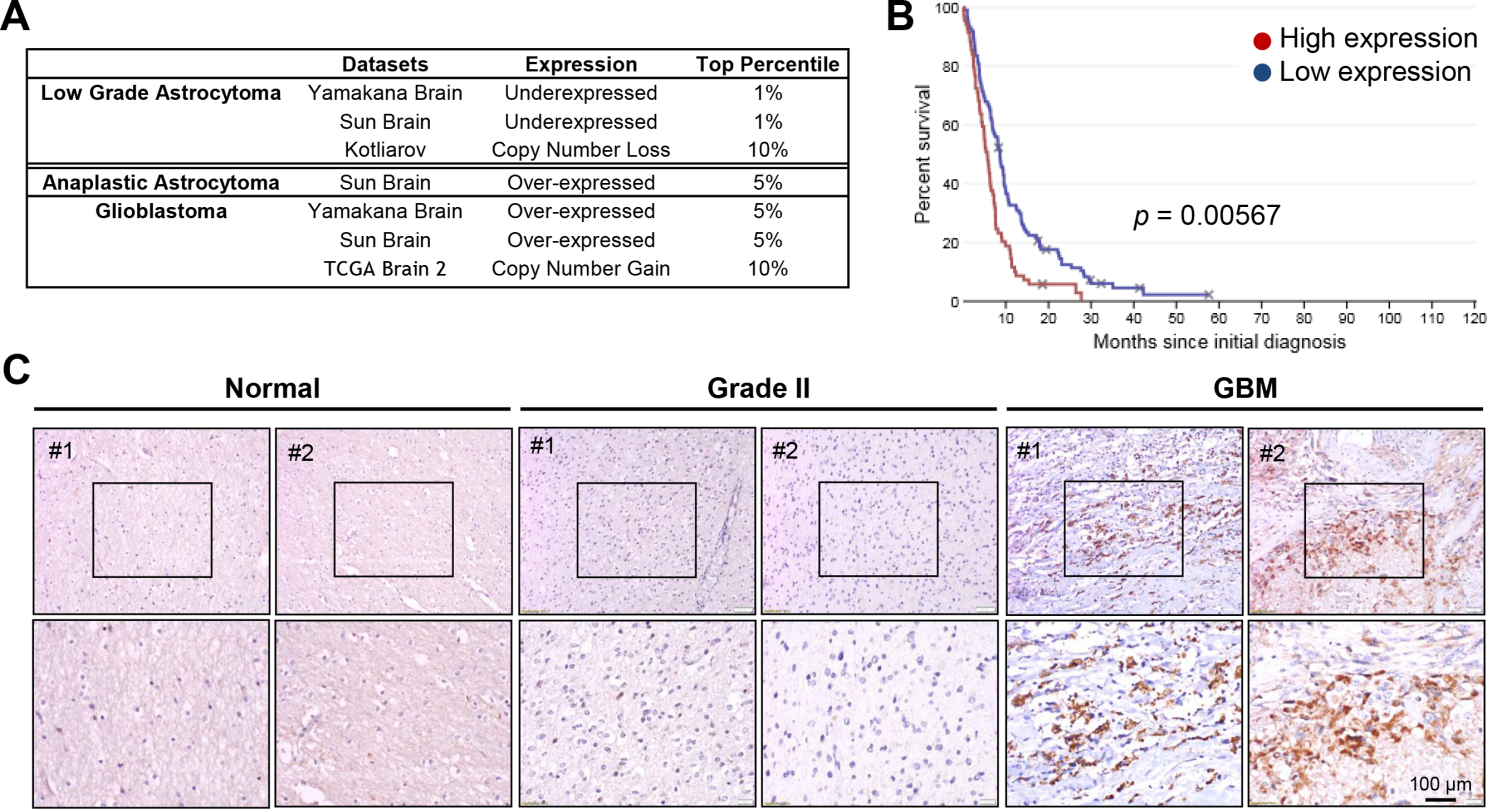
Lon is over-expressed in human malignant gliomas Data mining of previously published microarrays for Lon expression was performed using the: **A.** Oncomine database assessing Lon Expression in Astrocytic Tumors *vs.* Normal Brain. All studies identified showed significant differences between the normal brain and WHO grade II, III and IV astrocytic tumors. **B.** Rembrandt database examining Lon expression in human GBM patients correlated to survival. Kaplan-Meier Survival plot depicting survival of all GBM patients (n=178 patients) with Lon high expression (red line), and low expression (blue line). **C.** Immunohistochemical staining for LONP1 in human normal brains, Grade II and GBM surgical biopsies.

To investigate abnormalities in Lon expression in gliomas as compared with normal brain tissue, we used Oncomine to analyze all previously published relevant microarray data [20]. *Lon* mRNA levels were consistently higher in the anaplastic astrocytoma (World Health Organization grade III) and glioblastoma (World Health Organization grade IV, GBM) tumors as compared to the normal brain. Additionally, a copy number gain was seen in the GBM tumors (Figure 1A). In contrast, lower Lon levels were observed in low-grade astrocytomas (LGA), suggesting that the transition between low-grade to high-grade astrocytomas might be associated with Lon-mediated mitochondrial changes.

This intriguing result leads to the obvious question of whether patient survival may be affected by Lon over-expression. Using the Rembrandt database [21], we investigated once again whether previously published microarray data of gliomas (with accompanying survival outcome data) could answer this question *in silico*. A Kaplan-Meier Survival plot shown in Figure 1B clearly showed that high levels of Lon were associated with a significant decrease in patient survival.

Next, immunohistochemical analysis of Lon was performed in our laboratory using patient-derived glioma samples (Figure 1C). High Lon expression was detected in GBM tissues (especially in the areas of pseudo-palisading necrosis), but much lower expression was noted in Grade II tissues and normal brains (representative results shown). High Lon expression was also detected in tumor sections from nude mice subcutaneously injected with D-54 xenograft, as indicated by the abundant dark-brown staining (Supplementary Figure S1, maximum Lon staining seen in the areas of pseudo-palisading necrosis).

### Lon expression is induced by a variety of stressors and modulated by HIF-1α

**Figure 2:**
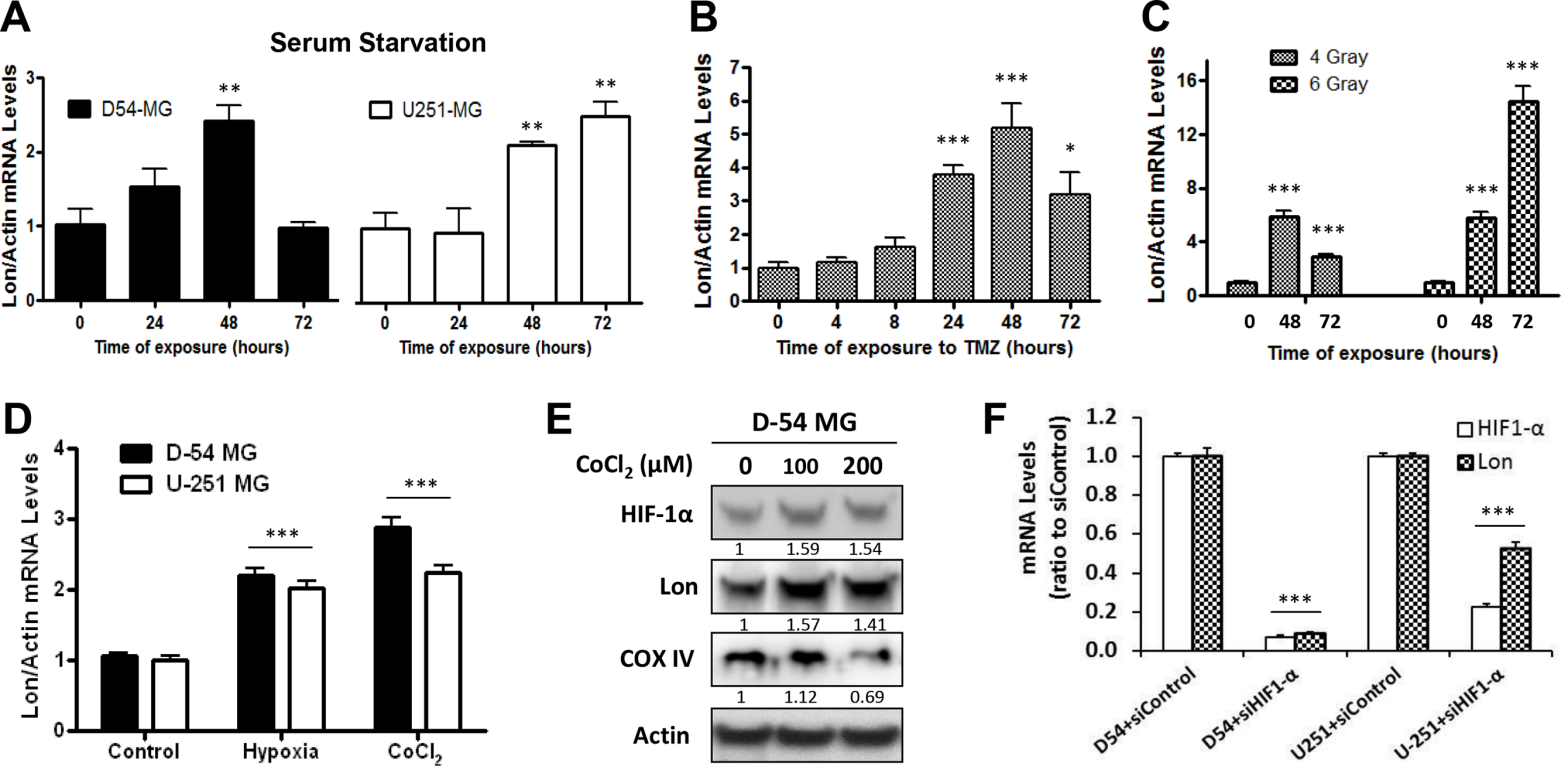
*Lon* expression is induced by a variety of stressors **A.** The normal culture medium (10% FBS) of D-54 and U-251 cells were replaced by serum-free medium for 3 hours. The cells were then allowed to recover in normal medium for the amount of time indicated. **B.** D-54 cells were treated with TMZ (500 μM). **C.** D-54 cells were exposed to 4 or 6 Gy of irradiation. **D.** D-54 and U-251 cells were cultured in low-oxygen concentrations (1%) or chemically-induced hypoxia (200 μM cobalt chloride) for 24 hours. Cells were collected at the indicated time points and RNA was extracted. qRT-PCR was then performed to measure the Lon mRNA levels. The relative expression levels were normalized by *ACTB*. **E.** D-54 cells were treated with 100μM or 200μM of CoCl_2_ for 24 hours. Western blot was used to detect HIF-1α, LONP1 and COX IV. Actin was used as the internal control. **F.** Treatment with four different siRNA targeted against the HIF-1α sequence leads to 80-90% decrease of *HIF-1α* mRNA levels as compared with siControl-treated cells, 72 hours after transfection. Treatment of cells with siRNA targeting *HIF-10α* reduced *Lon* mRNA levels (~80% in D-54 and ~50% in U-251), representative data showed. *p < 0.05, **p < 0.01, ***p < 0.001.

The Lon protease is a stress-responsive protein that is induced by multiple stressors [22, 23]. To examine changes in Lon expression following exposure to stressful conditions in glioma, we exposed two malignant glioma cell lines, D-54 MG and U-251 MG to a variety of stressors: serum starvation (Figure 2A), the most common chemotherapy drug used for GBM treatment - temozolomide (TMZ) (Figure 2B), irradiation (Figure 2C), and low-oxygen concentrations (1%) or 200 μM cobalt chloride (CoCl_2_) to induce hypoxia (Figure 2D). These conditions induced a significant increase in *Lon* mRNA levels in both D-54 and U-251 cells. The increase in *Lon* mRNA levels was accompanied by an accumulation of Lon protein in D-54 cells following 24 hour exposure to CoCl_2_ (Figure 2E). Exposure of cells to CoCl_2_ also resulted in increased HIF-1α protein expression and a dramatic reduction in COX IV protein levels, a known LON substrate (Figure 2E).

To examine if the ability of HIF-1α to modulate Lon expression is preserved in malignant glioma cells, we transfected both D-54 and U-251 cells with siRNAs (4 different constructs) directed against *HIF-1α* or a scrambled siRNA control. Three days after transfection, D-54 and the U-251 cells treated with siHIF-1α had an 80-90% reduction in *HIF-1α* mRNA expression as compared to the control-treated cells (Figure 2F, representative data included). Meanwhile, *Lon* mRNA levels in D-54 cells treated with the *HIF-1α* siRNA were eight to ten times lower than in the corresponding controls (representative data included). Similar results were found in U-251 cells. These results support the hypothesis that HIF-1α closely regulates Lon expression.

### Successful down-regulation of Lon in malignant glioma cells

**Figure 3:**
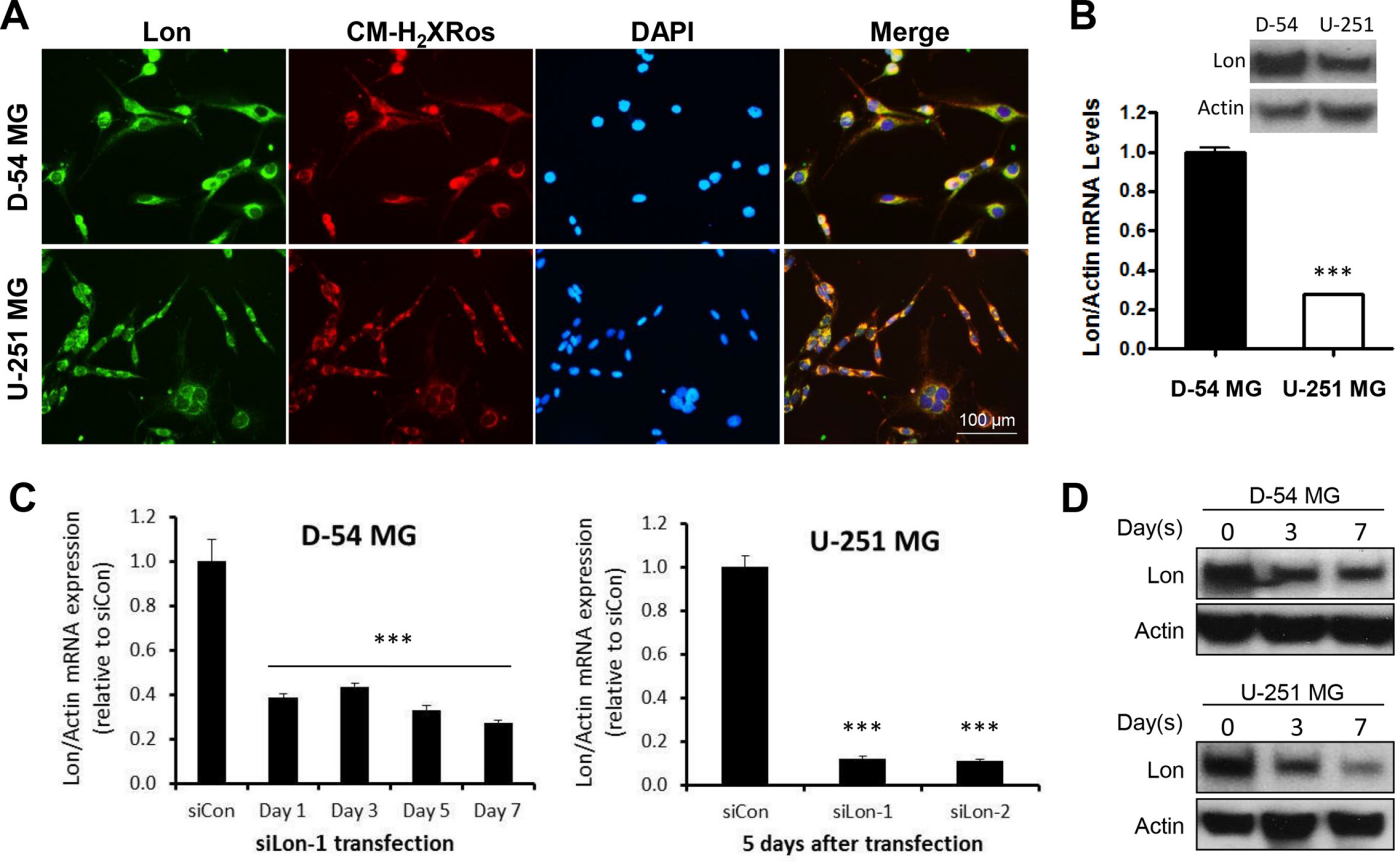
Successful knockdown of Lon in glioma cells **A.** Immunofluorescent staining for LONP1 (green) and CM-H_2_XRos (red) in D-54 and U-251 cells. Nuclei were counterstained with DAPI (blue). **B.** D-54 cells had a higher Lon expression than U-251 cells at both protein and mRNA levels. **C.** Successful knockdown of *Lon* mRNA was detected in D-54 cells even at Day 7 after *si*Lon-1 transfection. S*i*RNAs targeting *Lon* (*si*Lon-1and *si*Lon-2) significantly decreased *Lon* mRNA levels in U-251 cells 5 days after transfection. **D.** D-54 and U-251 cells were transfected by *si*Lon-1. Cells were collected and Western blot was used to detect LONP1 at 3 and 7 days after transfection.

Using immunofluorescent staining, we first identified the cellular localization of Lon in malignant glioma cells (Figure 3A). Lon positive staining (green) mainly co-localized to the mitochondria indicated by MitoTracker Red CM-H_2_XRos staining (red). Since our data showed that Lon was over-expressed in high-grade gliomas, and the Lon protease is up-regulated during conditions of high cellular stress [23], we next sought to investigate the role of Lon in glioma by knocking down Lon expression. We first evaluated the basal expression levels of Lon in D-54 and U-251 cells, and found that Lon is more highly expressed in D-54 cells than in U-251 cells at the mRNA and protein level (Figure 3B). Using siRNA targeting Lon, we successfully knocked-down Lon expression in D-54 and U-251 cells, and the transfection efficiency was identified by quantitative RT-PCT (Figure 3C, Supplementary Figure S2) and Western blot (Figure 3D). The Lon down-regulation lasted 7 days after transfection.

### In malignant glioma cell lines, Lon expression is required for survival in hypoxic conditions, and functional mitochondrial respiration

**Figure 4:**
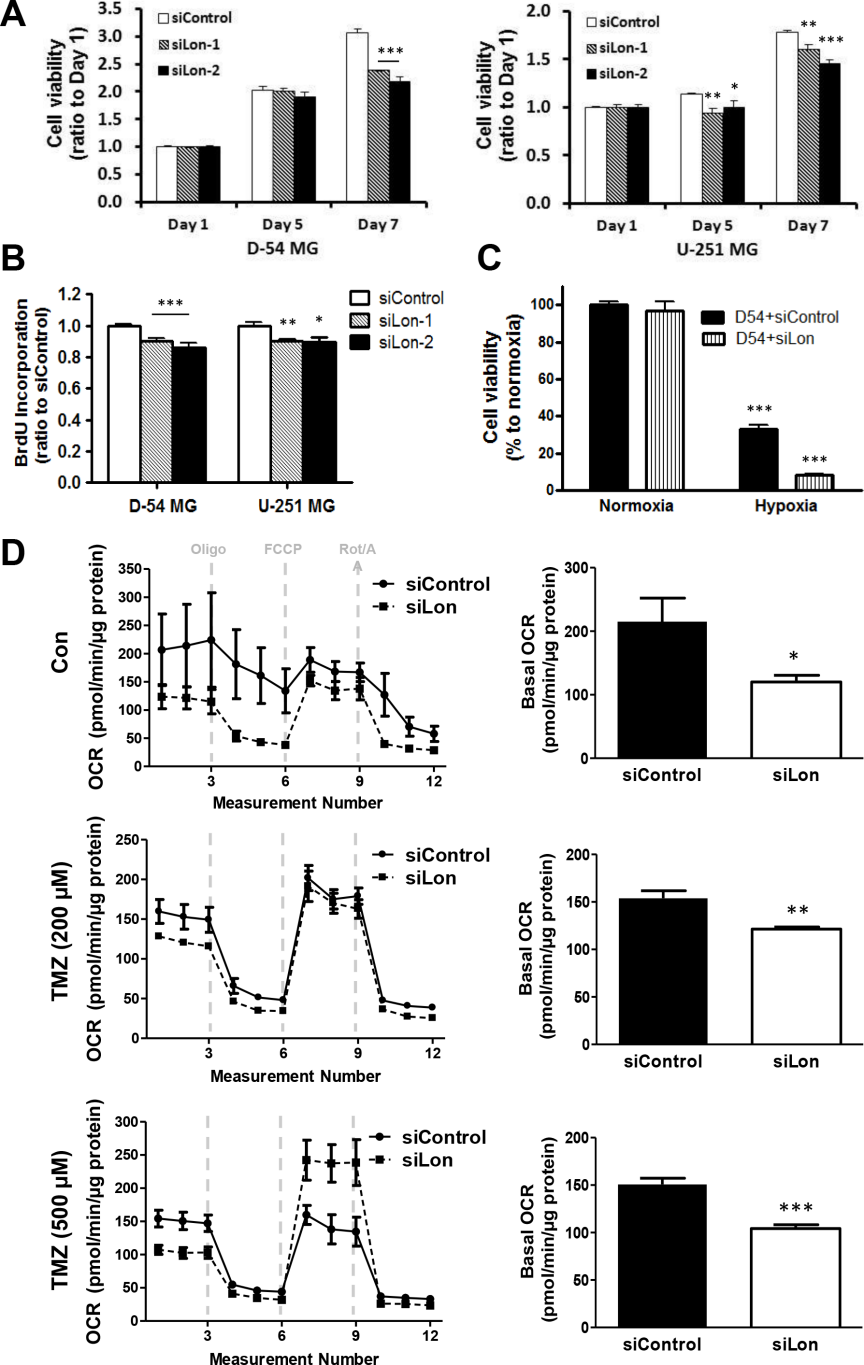
Effect of Lon knockdown on cell proliferation and mitochondrial respiration in glioma cells **A.** The growth rate of D-54 (left panel) and U-251 (right panel) cells transfected with *si*Control, *si*Lon-1, and *si*Lon-2 was measured by MTT assay. Data are presented as the ratio of the absorbance of transfected cells to that of untransfected (baseline) cells. **B.** The effect of Lon knockdown on cell proliferation was measured by BrdU incorporation analysis. **C.** D-54 cells were transfected with *si*Lon-1. After 24 hours, cells were then subjected to hypoxia (1% O_2_) for 48 hours, and cell viability was measured by MTT assay. **D.** Mitochondrial respiratory profile of D-54 cells transfected with *si*Lon-1. Twenty-four hours after transfection, cells were plated on Seahorse XF24 cell culture microplates, and treated with 200 μM or 500 μM of TMZ for 5 days. After establishing a basal OCR, oligomycin (2 μM), FCCP (0.5 μM), and rotenone (1 μM) plus antimycin A (1 μM) were sequentially added (left panel). The basal OCR was calculated using the difference between baseline and oligomycin treatment (right panel). Data graphed as mean ± SEM, n=3 per treatment group. *p < 0.05, **p < 0.01, ***p < 0.001.

We next investigated if Lon is required for survival in malignant glioma cells. Decrease in viability of cells treated with siLon as compared to siControl was observed on Day 5 and/or 7 after transfection (Figure 4A), indicating that Lon may modulate glioma cell proliferation under normoxic conditions. The effect of Lon knockdown on cell growth was further measured by BrdU incorporation analysis (Figure 4B). In addition, only 8% of the Lon knockdown cells survived as compared with 33% of the control group following 48 hours of 1% O_2_ culture condition (Figure 4C). Thus, Lon down-regulation not only leads to impaired cell viability under normoxic conditions, but makes cells much more sensitive to hypoxia, resulting in a four-fold decrease in viability under hypoxic conditions.

As one of the mitochondrial matrix proteases, Lon play a critical role in regulating aerobic respiratory function. To investigate the effect of Lon knockdown on the cellular bioenergetics of glioma cells, we assessed the basal cellular oxygen consumption rate (OCR) using the Seahorse Biosciences Extracellular Flux Analyzer. OCR is a measure of oxidative phosphorylation (OXPHOS) and is indicative of mitochondrial respiration. We found that basal OCR was considerably lower in siLon group as compared with that of siControl group (*p*<0.05, Figure 4D, upper panel), reflecting a decrease in respiratory function of glioma cells due to Lon knockdown. Meanwhile, TMZ treatment resulted in a further marked reduction of OCR (Figure 4D, middle & lower panels).

### Lon inhibitor CC4 inhibits cell growth and synergistically enhances the therapeutic efficacy of chemotherapy drugs

**Figure 5:**
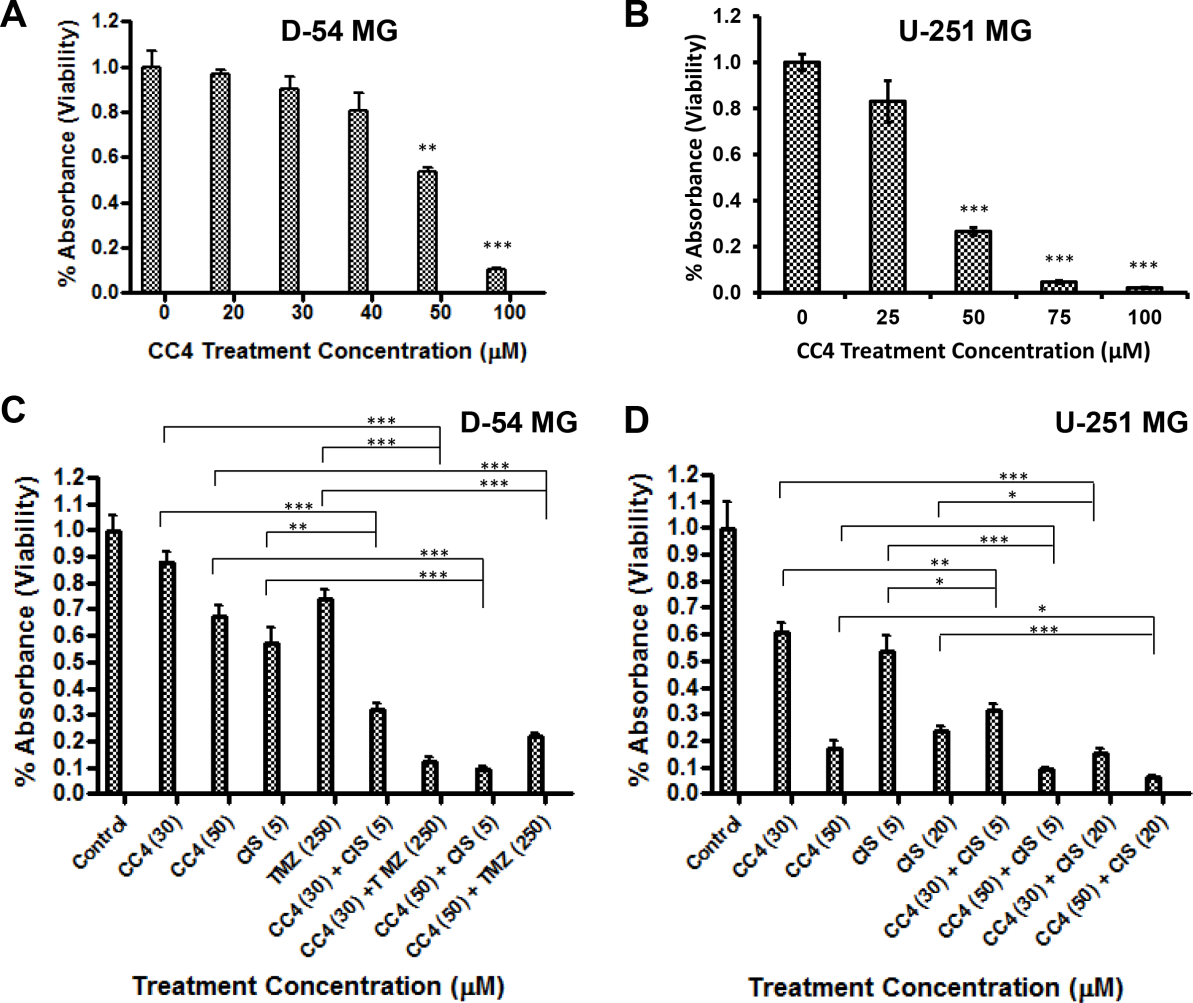
Lon inhibitor CC4 inhibits cell growth and synergistically enhances the therapeutic efficacy of chemotherapy drugs D-54 **A.** and U-251 **B.** cells were incubated for 3 days with increasing concentrations of CC4. D-54 **C.** and U-251 **D.** cells were incubated with TMZ and/or Cisplatin and CC4 for 72 hours. Cell viability was measured by MTT assay. The relative numbers of proliferating cells compared with control are presented as the mean ± SEM. *p < 0.05, **p < 0.01, ***p < 0.001.

Since our results revealed an antitumor effect of Lon knockdown, we next investigated the efficacy of the Lon inhibitor – CC4, which is one of a series of coumarinic compounds able to selectively inhibit Lon serine protease activity [24] in glioma cells. As shown in Figure 5A & 5B, CC4 significantly inhibited the growth of D-54 and U-251 cells in a dose-dependent manner, suggesting that CC4 may serve as a lead compound in the development of specific and efficient inhibitors of Lon protease as potential cancer treatments.

To examine the potential usefulness of a combinatory approach: Lon inhibition (CC4) and chemotherapy drug exposure (TMZ and cisplatin), D-54 (Figure 5C) and U-251 (Figure 5D) cells were treated with varying concentrations of TMZ and/or cisplatin with or without 30 μM or 50 μM of CC4. While TMZ and cisplatin repressed the growth of glioma cells in a dose-dependent manner, the addition of CC4 further potentiated the inhibitory effects of TMZ and cisplatin, indicating that the combination of CC4 and chemotherapy drugs produces a synergistic inhibition.

## DISCUSSION

The importance of mitochondrial dysfunction in cancer cell adaptation to hypoxia is an important yet under-researched topic. Developing tumors inevitably deplete their oxygen and nutrient supplies, thus only tumor cells which adapt to hypoxia, survive and become more aggressive/invasive malignancies [3]. Glioblastoma is a tumor characterized by extensive hypoxia-induced phenotypic changes such as abnormal vascular proliferation and necrosis [25, 26]. Previous studies have shown that hypoxic adaptation is a prerequisite for glial tumor progression and that the degree of hypoxia correlates with tumor grade, increased risk of recurrence after surgery and clinical aggressiveness in glioma patients [27]. However, relatively little is known regarding the underlying mechanism(s) linking the degree of hypoxia to poor prognosis. The transcription factor HIF-1α has emerged as a key regulator of oxygen homeostasis in malignant glioma and its expression correlates with a malignant glioma phenotype in patient samples [28]. While HIF-1α is involved in a plethora of pathways, only a few genes have been established as direct targets by identification of critical HIF-1α binding sites [29]. Herein, we demonstrate that Lon is an HIF-1α target and plays an important role in the malignant glioma phenotype.

Our investigation began with an in-depth analysis of previously reported microarray data from human glial tumors. Mining the major brain tumor databases accessible on the Oncomine service, we show that Lon expression correlates with the grade of glial astrocytic tumors. Akin to HIF-1α, high Lon expression correlated with high-grade glioma tumors (Figure 1A). To determine if this relationship translated to poorer outcome for patients with glioma, we mined the Rembrandt database. Interestingly, patients with gliomas presenting elevated Lon expression suffered a significant reduction in survival (Figure 1B). Moreover, immunohistochemical analysis clearly displayed a strong expression of Lon in GBM tissue sections – and especially in the hypoxic areas, but much less in Grade II tissues and normal brains (Figure 1C). Taken together, expression levels of Lon clearly correlate to tumor grade and poorer outcome in human glioma.

It has been reported that Lon is up-regulated under various stress conditions [22, 23]. Our data demonstrated that *Lon* mRNA and protein expression was promptly induced in response to a variety of stimuli, such as serum starvation, drug treatment, irradiation, and hypoxia (Figure 2A-2E). In addition, direct knockdown of *HIF-1α* using siRNA resulted in the abrupt decrease of *Lon* mRNA expression (Figure 2F), confirming that HIF-1α effectively controls Lon expression in malignant glioma lines as was previously shown in normal cell lines [6].

As one of the mitochondrial matrix proteases, we identified the cellular localization of Lon mainly localized to mitochondria in glioma cells (Figure 3A). Next, the direct role of Lon in glioma cells was examined by knocking down Lon expression through RNA interference (Figure 3C, 3D). Inhibition of Lon blocked glioma cell proliferation under normoxic conditions (Figure 4A, 4B), which is consistent with previous findings in bladder cancer [30], colon cancer [31], and cervical cancer cells [32]. Moreover, malignant glioma cells not only over-express Lon in hypoxic conditions, but require Lon expression to survive hypoxia. Only 30% of the control D-54 cells survived when subjected to low-oxygen environment (1%) for two days. However, if the same cells were pretreated with Lon *si*RNA precluding Lon induction, only 8% of the D54-MG cells survived (a four-fold difference) (Figure 4C), indicating that Lon plays a critical role in regulating aerobic respiratory function. Our data showed that Lon knockdown in glioma cells resulted in impaired respiratory function as indicated by decreased OCR. Meanwhile, the chemotherapy drug TMZ resulted in a further marked reduction of OCR (Figure 4D). These results demonstrate that Lon enables the mitochondria to more efficiently function in response to hypoxic conditions, which are common characteristics of the tumor microenvironment.

Our data shows that Lon was over-expressed in glioma tissues, while inhibition of Lon blocked glioma cell proliferation. This suggests that Lon may serve as a potential therapeutic target in gliomas. In our study, using a specific Lon inhibitor CC4, we demonstrated that CC4 not only significantly suppressed glioma cell growth, but further potentiated the antitumor effects of two commonly used chemotherapy drugs with cross the blood-brain barrier and have proven activity in brain malignancies, TMZ and cisplatin (Figure 5).

In summary, we demonstrate that Lon is integrally involved in hypoxic adaptation and malignant glioma progression. We propose a model of glioma progression in which hypoxia stabilizes HIF-1α protein, which in turn binds to the Lon promoter and promotes Lon expression. Next, Lon modulates mitochondrial metabolism and maintains mitochondrial biogenesis in the changed environment, making the glioma cell increasingly able to survive in low-oxygen conditions. Pharmacological inhibition of HIF-1α has been tried in a multitude of cancers with limited success [33], most likely due to the complexity of the HIF-1α transcriptome, which contains not only genes that promote tumor cell survival but also genes that promote growth arrest [34] and tumor cell death [35]. As Lon is required for metabolic adaptation to hypoxia but has a smaller and well-defined number of possible targets, its inhibition might prove to be a valuable target for future cancer treatments in hypoxic tumors, which are very resistant to traditional treatments.

## MATERIALS AND METHODS

### Chemicals and reagents

All standard chemicals, buffers and reagents, unless otherwise indicated, were purchased from Sigma Aldrich. Lon inhibitor CC4 (Coumarinic Compound 4) was synthesized by Dr. Chris Vanderwal from Department of Chemistry, UC Irvine according to published data [24].

### Glioma tissues and cells

Institutional Review Board approval was obtained at University of California Irvine Medical Center. Surgical specimens of brain tumors were obtained from patients who had undergone tumor resection with the neuropathological review completed by a specialized neuropathologist. The established malignant human glioma cell lines, U-251 MG and D-54 MG, were maintained in DMEM/F-12 medium containing 292 μg/ml glutamine, 1% penicillin/streptomycin and 10% FBS (Omega Scientific, Inc.). All cells were cultured at 37°C in a humidified incubator with 5% CO_2_.

### Oncomine database

The use of the database is described previously [20]. All data sets including astrocytic tumors were selected.

### Rembrandt database

In 2005 the National Cancer Institute and the National Institute of Neurological Disorders and Stroke (NINDS) initiated the REMBRANDT (REpository for Molecular BRAin Neoplasia DaTa (http://www.betastasis.com/glioma/rembrandt/) database [21]. Database consists of ~524 gene expression arrays from human gliomas.

### Immunohistochemical and immunofluorescent staining

VECTASTAIN ABC kit and DAB substrate kit (Vector Laboratories, Inc.) were used for immunohistochemical staining. Formalin-fixed, paraffin-embedded tissue sections (5 μm) were deparaffinized and rehydrated. After antigen retrieval, the endogenous peroxidase activity was blocked with 1% H_2_O_2_ in PBS for 20 min. The sections were incubated with 5% normal goat serum and then exposed to rabbit anti-LONP1 antibody (15440-1-AP, 1:50, ProteinTech Group, Inc.) at 4 °C overnight, followed by incubation with biotinylated anti-rabbit IgG for 1 hour at room temperature. The sections were incubated with ABC reagent and then DAB substrate solution until desired stain intensity developed. Hematoxylin was used as counterstain. Slides were then mounted with Permount (Fisher Scientific).

For immunofluorescent double staining, live cells were first incubated with MitoTracker Red CM-H_2_XRos (Invitrogen) at 500 nM for 45 min at 37°C. The cells were then fixed and incubated with anti-LONP1 antibody, followed by FITC-conjugated anti-rabbit secondary antibody (Millipore). Slides were mounted with VECTASHIELD Antifade Mounting Medium with DAPI (Vector Laboratories, Inc.). Fluorescent images were generated using the Nikon Ti-E inverted microscope with a 20x objective (NA 0.75).

### Quantitative RT-PCR (qRT-PCR) analysis

Total RNA was extracted using RNeasy Mini Kit (Qiagen), and cDNA was generated using the iScript™ cDNA Synthesis Kit (Bio-rad). Quantitative PCR reactions (iQ™ SYBR Green Supermix, Bio-rad) were conducted using a Bio-Rad CFX96 Real-time System, and the gene expression levels were normalized to those of *ACTB*. The primers used are the following: Lon forward CAAGGTGCTGTTCATCTGCA and reverse AGCTTGGCCTTGCTCTCATC, HIF-1α forward TACTAGTGCCACATCATCAC and reverse TTCGCTTTCTCTGAGCATTC (IDT, Integrated Device Technology). *ACTB* (QT00095431) was purchased from Qiagen.

### Western blotting

Antibodies used were LONP1 antibody (15440-1-AP, ProteinTech Group, Inc.), HIF-1α (GTX127309, GeneTex), COX IV (ab62164, Abcam) and β-actin (NB600-501, Novus). The images were exposed by KODAK M35A X-OMAT Processor.

### siRNA transfection

Four human FlexiTube siRNAs targeting *LONP1* (SI00068488, SI00068495, SI00068509 and SI03097297) and negative control siRNA (1022076) were purchased from Qiagen, and were transfected into cells using HiPerFect transfection reagent (Qiagen). One siLONP1 (SI00068488, siLon-1) was used in most of experiments unless otherwise indicated. Down-regulation of Lon was confirmed by RT-PCR and Western Blotting. A kit of siRNAs targeting *HIF-1α* (GS3091) was purchased from Qiagen.

### Cell viability

The cells were seeded at approximately 1×10^4^/well in a final volume of 200 μl in 96-well microtiter plates. The plates were incubated at 37°C under normal or hypoxic conditions (1% O_2_) or exposed to various concentrations of drugs as indicated. Cell viability was determined using the Cell Viability Kit II (XTT) (Roche Applied Science).

### BrdU cell proliferation analysis

Cell proliferation rates were determined by BrdU incorporation according to the manufacture's recommendation (Calbiochem). Briefly, the cells were incubated with BrdU Label for 24 hours, and then fixed. Monoclonal anti-BrdU antibody was added, followed by peroxidase-conjugated secondary antibody. The BrdU incorporation was measured at dual wavelengths of 450 and 570 nm, using a Model 680 Microplate Reader (Bio-Rad).

### Seahorse XF24 metabolic flux analysis

Mitochondrial respiratory function was assessed using the Seahorse XF24 Extracellular Flux Analyzer. Oxygen consumption rates (OCR) were measured using the Cell Mito Stress Kit (Seahorse Bioscience, Billerica, MA). The cells (7.5×10^4^/well) were plated on Seahorse XF24 cell culture microplates. Baseline rates were measured at 37°C three times before the sequential injection of the following mitochondrial inhibitors: oligomycin (2 μM), triflouromethoxyphenylhydrazone (FCCP, 0.5 μM), and lastly, rotenone (1 μM) and antimycin A (1 μM). Three measurements were taken after addition of each inhibitor. All measurements were normalized to protein content per well using a Qubit 2.0 fluorometer (Invitrogen, Grand Island, NY). OCR data was collected using the XFe Wave software (Seahorse Bioscience), and analyzed using GraphPad Prism 5 (GraphPad Software, La Jolla, CA, USA). Statistical significance was measured by student's paired *t*-test.

### Statistical analysis

Statistical analyses were performed and graphs generated using Prism 5 (GraphPad). All values were presented as mean ± standard error of the mean (S.E.M.) when at least 3 observations were available. Statistical significance was measured by unpaired *t*-tests or one-way ANOVA unless otherwise indicated.

## SUPPLEMENTARY MATERIALS FIGURES


