# Supplementary material for: Mitochondrial Lon is over-expressed in high-grade gliomas, and mediates hypoxic adaptation: potential role of Lon as a therapeutic target in glioma
Source: Oncotarget. 2016 Oct 15;7(47):77457–67. doi: 10.18632/oncotarget.12681 (PMC5340227; doi:10.18632/oncotarget.12681)
Supplement: Supplementary file 1 [file oncotarget-07-77457-s001.pdf]

Mitochondrial Lon is over-expressed in high-grade gliomas, and mediates hypoxic adaptation: potential role of Lon as a therapeutic target in glioma

SUPPLEMENTARY FIGURES

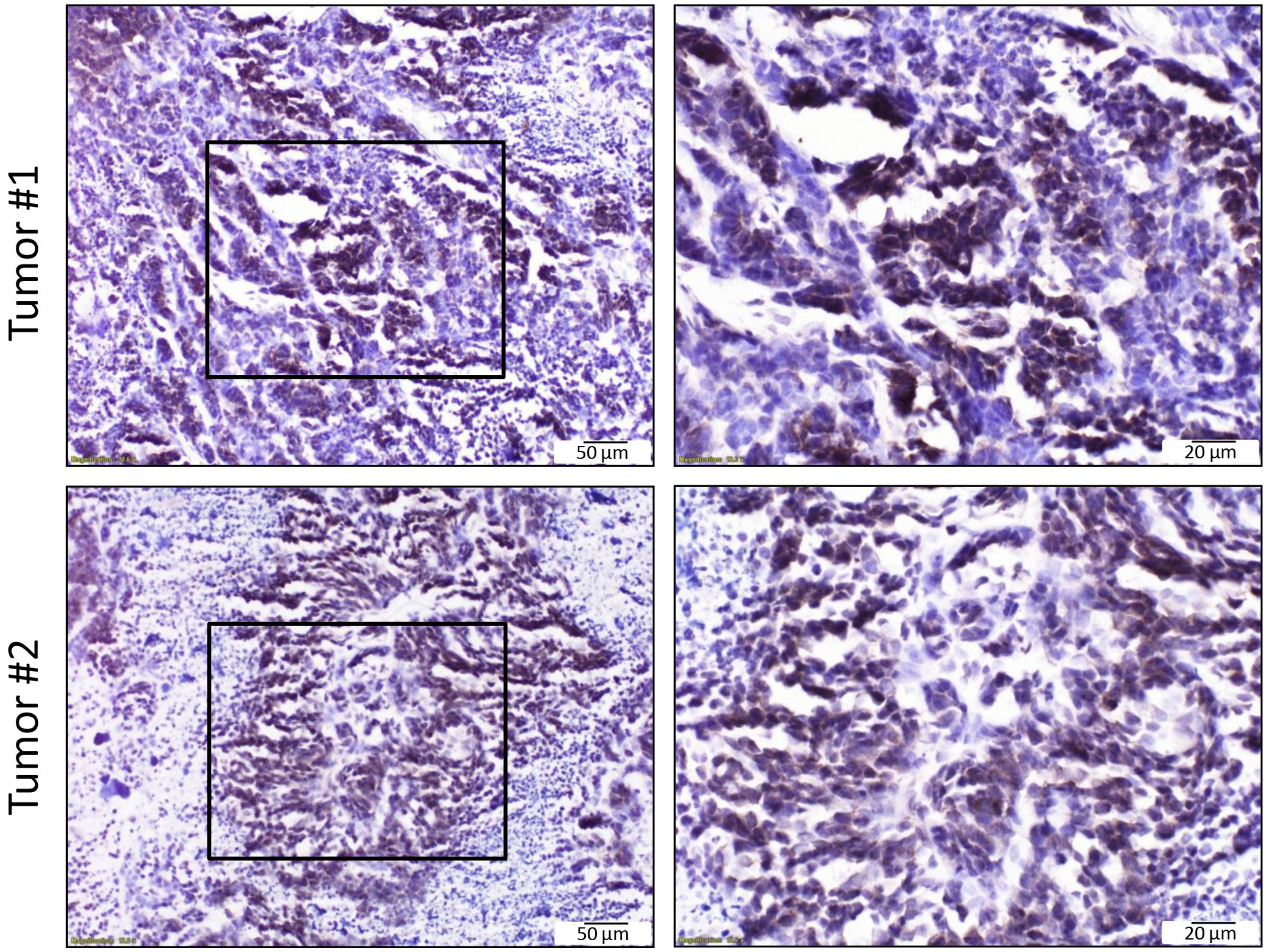

Supplementary Figure S1: Representative immunostaining of Lon in D-54 MG xenografts. D-54 xenografts were subcutaneously injected into nude mice anterior to their thighs. Lon staining was performed using paraffin-embedded sections from tumor mass of xenografts. Microscopic magnification was x200 (left) and x400 (right).

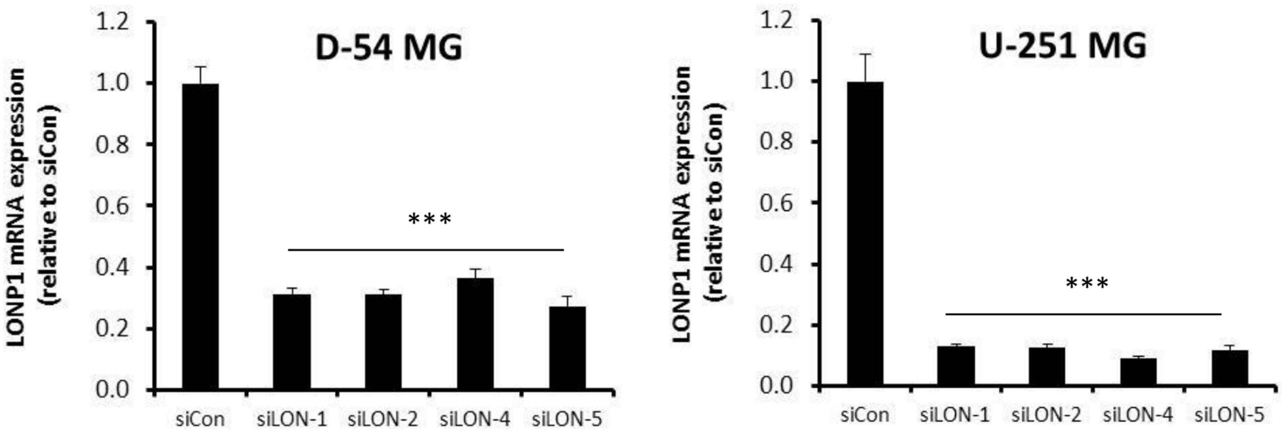

Supplementary Figure S2: Successful knockdown of *LONP1* mRNA expression in glioma cells. Four human FlexiTube *si*RNAs targeting *LONP1* and negative control *si*RNA were transfected into cells for 48 hours.
